# The Affective Creativity of a Couple in Dementia Care

**DOI:** 10.1007/s11013-019-09662-5

**Published:** 2019-11-11

**Authors:** Jong-min Jeong

**Affiliations:** Department of Social Anthropology, University of Manchester, Oxford Road, Manchester, M13 9PL UK

**Keywords:** Couple, Affective practice, Creativity, Arts activities, Feeling body, Co-production

## Abstract

The capacity to feel and express themselves in response to worldly surroundings is a defining feature of who a person living with dementia is, and can have profound effects on the ways in which they think, act and express creativity. Drawing on a year of intensive collaborative work with residents living with dementia in an Orthodox Jewish care home in London, I extend our perceptions and understandings of how a couple experiences their day-to-day lives, with particular attention paid to their affective practice in creativity. I demonstrate how the affective creativity of the couple emerges, circulates, and transforms as a spouse’s dementia develops, whilst feeling bodies continuously (re)make relations and familiarize themselves with the immediate surroundings through the making of artworks.

## Introduction

**Fig. 1:**
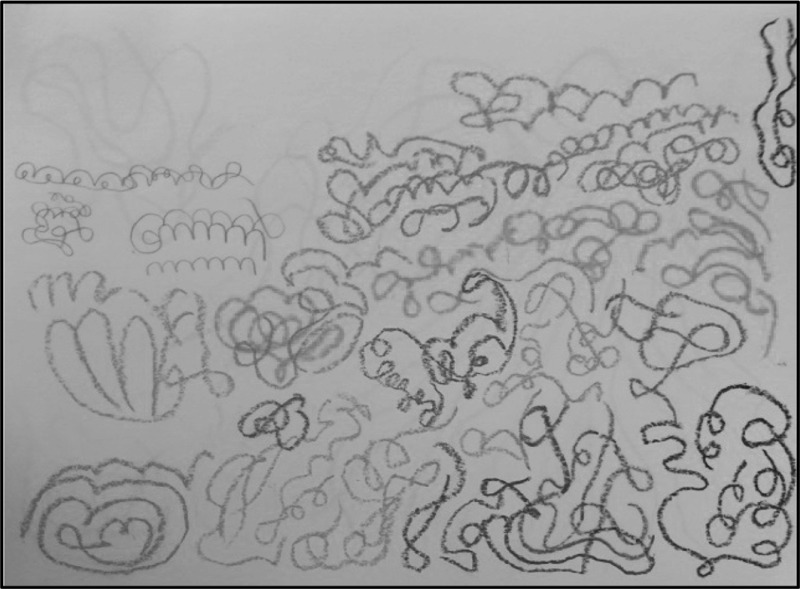
Rachel’s Painting

Rachel (87), living with moderate dementia, draws many geometrical and abstract figures, examining them in detail, inch by inch, over the rim of her glasses in the arts and crafts center at an Orthodox Jewish care home (Home) in London. Jacob (86), her husband, who does not have dementia, also draws a still life at a distance from her seat, often looking over at what she is doing. For her, time seems to flow extremely slowly as all her attention is focused on her drawing, which takes the whole morning around from 10 am to 11:45. She was offered a cup of coffee with one spoonful of sugar and a piece of cake during the morning tea break at about 11 am. Activity leaders and I tell her to drink it before it gets cold, but she does not seem to care. Only after she finishes her artwork does she have the cold coffee and cake. Jacob approaches and stands by her looking at the drawing and her face in turn. Adjusting her glasses, Rachel looks at her husband with a smile. He may wish to know what and how she thinks about her artwork, but he does not ask or say anything; instead they just exchange a light smile. The style of her artwork is uncanny (Fig. 1): parts appear to follow a rule of repetition, yet, seen as a panorama, it shows a totally different composition and a harmony. Some residents say that there are beautiful combinations of different colors, others just shrug as if they do not understand what she is trying to draw. Activity leaders praise her “lively”, “repetitive,” “colorful,” and “harmonious” work, expecting the couple to join in the activity more often.

These days, Rachel’s craftwork, painting, and drawing have changed dramatically, from the figurative and representational to the abstract. Since the couple moved into the Home in 2013, Rachel’s dramatic transformation in her modes of expression has reflected significant changes in her dementia (Mendez 2004). With recent developments in neuroscience, we can, to some extent, define how the brain interacts, translates, and transforms visual-spatial perception, language and aesthetics (Rose 2004). This scientific achievement enables us to identify the neurological deterioration of the brain through art, by tracing the transformations of expression in terms of tone, texture, color, brightness and abstractness over a substantial period of time (Rustin 2008). Yet, what draws more attention at the Home from her works are the ways in which Rachel and Jacob “do things together” in the making of couplehood and artworks *with* and *through* her loss (Hellström, Nolan, and Lundh 2005).

Drawing on arts practice-based collaborative ethnographic research in the Home, I reveal how the feeling bodies and affective practices of couples have an impact on the experience of creativity, and at the same time, the ways in which the processes and the outcomes of artworks transform the affective dimensions of couplehood. Consequently, I argue that it is not sufficient to describe the creativity of couples as merely an outcome of a purely mental and cognitive process that takes place in an individual’s brain, or as an individual’s genuine artistic skills that the artist still has; rather it emerges in the process of collaborative work by distributing affective creative affordance between people, things, and the environment, as a collaborative, improvisational and relational social phenomenon, or what I call the “affective dimension of creativity.”

## Arts, Affective Creativity and Couples

There has been a growing recognition from policy makers, researchers, caregivers and practitioners in dementia that the arts can not only offer biomedical and therapeutic benefits (Chancellor, Duncan, and Chatterjee 2014; Gretton and ffytche 2014; de Medeiros and Basting 2013; Selberg 2015; Willis 2014). It also can improve quality of life and self-care including emotional, social, and spiritual wellbeing (Basting 2006, 2014, 2017; Hannemann 2006; Hayes and Povey 2011; Schneider 2018). In particular, researchers have been asked to pay attention to arts activities in order to reveal the remaining potential artistic capacity of people living with dementia (Hannemann 2006; Palmiero, Di Giacomo, and Passafiume 2012).

However, most of the aforementioned studies are focused more on the biomedical, psychosocial and therapeutic “product” of the-moment-of-engagement, such as relieving and managing dementia-related symptoms, but regrettably less on the “process” (Beard 2011:645); namely the arts become recognized merely as therapies or biomedical signatures of cognition and brain atrophy (Mendez 2004; Rose 2004), and thus the ways in which artistic creativity emerge, engage, and interact remain unexplored. This is largely caused by the methodological and environmental conditions of research. Those above-mentioned interventions are predominantly conducted in controlled environments in line with detached observations, interviews and surveys in order to precisely measure and analyze the cause and effect of the experiments. It is no wonder that this outcome-centered approach exposes a significant gap in “the more mundane ordinary forms of creativity” (Bellass et al. 2019:2) “beyond the temporal boundaries” of the events (Gross et al. 2015:31).

Whereas there are a number of studies demonstrating the significance of shared activities and approaches towards couples’ experiences of dementia (Balfour 2014; DiLauro et al. 2015; Hydén and Nilsson 2013), few studies have been conducted from a relational perspective focusing on couplehood (Unadkat, Camic, and Vella-Burrows 2016). Although there has been a consistent and continuously increasing interest in joint or collective research participation and methodology for couples (e.g. Hellström, Nolan, and Lundh 2005, 2007; Hydén and Nilsson 2013; Molyneaux et al. 2011), these researches mainly rely on the perspectives, narratives and experiences of the spouses without dementia (e.g. Hill, Yeates, and Donovan 2018; Merrick, Camic, and O’Shaughnessy 2013, van Wijngaarden et al. 2018). Needless to say, artistic creativity has mainly been conceptualized, appreciated and facilitated by others, such as researchers or practitioners. Consequently, very little research has explored how those affected actually feel, appreciate, and perceive their artworks and creativity from a couples’ perspective. This lacuna shows a heed for more attention to be paid to the everyday artistic creativity of couples, focusing more on the process, context and voices of couples from a relational perspective. In this article, I re-evaluate what established studies of artistic creativity have accomplished and what they have focuses less on, and suggest how we could move forward from there, with particular attention to the affective dimension of artistic creativity of couples, based on the following questions:

- Do the feeling bodies and affective practices of couples limit or facilitate the exercise of artistic skills?
- How do the ordinary affective creativities of couples operate across day-to-day life beyond specifically designed activities?
- What impact, if any, does affective creativity have on a couplehood, or vice versa?
- How do couples co-construct their story of couplehood in the making of artworks?

Rather than discussing these questions from theoretical, therapeutic, or psychosocial perspectives, I combine them into an ethnographic investigation, exploring the ways in which affective creativity emerges, transforms and circulates among those involved, focusing particularly on one couple, Rachel and Jacob, in the Home.

Arts activities are diverse and thus it is difficult to define arts in dementia (Schneider 2018). Drawing on Anne Basting’s (2018) understanding of arts as “a way of being in relationship/bringing people into relationship” I use the term arts to mean participatory, expressive and performative activities and engagements that take place in everyday life in care home settings. The arts thus encompass visual arts, music, drama, dance/walking/body movement and writing, as well as manual arts and crafts such as crafting pottery and needlework. In addition, I regard the arts as a medium which offers a platform not only where those affected can express their interiority (Hannemann 2006), but also where various artworks are created, transformed and circulated. More importantly, I revalue the arts as a mundane way of creating social life, referring to the ways in which lived experiences of dementia emerge and become otherwise in the making of artworks, or what I call “dementia-becoming.”

Applying Margaret Wetherell’s (2012:96) concept of “affective practice” I regard affect as a kind of “assemblage” and “articulation” of embodied felt experiences and discursive practices (2013:351), rather than a kind of “beyond, below and past discourse” (Wetherell 2013:350), an extra-discursive event (Massumi 2002), or a non-representative domain/theory (Thrift 2008). Affective practice in this article thus implies not only a new epistemological framework, but also a methodological tool allowing us to explore the ways in which the feeling bodies and discursive practices of the couple encounter, engage, and resonate with other bodies, physical objects and material environments. Of course, I assume that as Rachel’s dementia progresses, her affective practices show more sensory, haptic, or kinaesthetic bodily movements, and less of the practices of the “speaking subject” (Blackman and Couze 2010:9).

Building upon Klee’s idea of “form-giving” (1961:457)—which brings something from absence into being—without falling into the temptation of simplifying the practice, Tim Ingold understands “making” as “a process of *growth*” and the maker as “a participant in amongst a world of active materials” (italics in original) (Ingold 2013:21). Applying Ingold’s understanding, creativity is not the final outcome of unprecedented ideas, nor does it start from absolutely nothing; rather, it is generated in the process of a continuous attending to the world in formation (Ingold 2014). It is not necessarily already given, but is rather the action of engaging with a world that we are reaching out to from the unknown, and thus there is no mastery (Ingold 2013). Here is the point where culturally improvised creativity plays a significant role in this form-giving process: although residents with dementia may retrieve and call upon (remaining) skills, memories and abilities that have been sedimented and embodied within them, they all, to some extent, have to improvise as they enter a world, involving the use of untrained, pre-reflexive, and/or socially embodied bodily apparatus (e.g. Kontos 2003). In this sense, engaging is a way of form-giving that always involves intersections, connections and encounters, alongside obstructing, avoiding and parting with others, things and the environment. These heterogeneous, enmeshed and contingent relations in the field of action generate new formations and conditions of the present in response to uncertain and undetermined circumstances (Deleuze and Guattari 2004). Drawing, for example, consists of particular kinds of social and creative components in practice that include materials, organizational bodies, staff, tools, environment, music, a wheelchair, coffee, biscuits, individual moods and residents, to name just a few in care home settings.

## Everyday Practice as a Research Method

The lack of a generally accepted methodology for studying the feeling bodies and affective practices of couples in dementia raises three practical and ethical problems for anthropological fieldwork, findings, and analysis;

- How can researchers perceive the extent to which affective practices are shaped and shape in response to their immediate surroundings?
- How can researchers access and obtain people’s affective practices whilst not necessarily intervening in their ordinary lives?
- How can researchers ensure the capacity of participants to consent to their participation throughout the course of the research?

Building upon my decade-long personal relationship with the Home, its residents and their significant others as a befriender-volunteer as well as researcher, I conducted an intensive ethnographic study of affective creativity as a part of my PhD project from May 2014 to May 2015. I regarded everyday practice as a research method, conducting fieldwork by talking, walking, eating, doing and acting together with residents and at the same time being befriended by residents. These approaches and procedures raise specific ethical concerns about participant recruitment and the research process in relation to the Mental Capacity Act (2005) in England and Wales. My research obtained ethical approval from the National Research Ethics Service Committee (14/NW/0054) in 2014. All identifiable subjects have been anonymized.

In more detail, recruitment was carried out through face-to-face contact, and I re-introduced myself as a PhD student to those who had known me as a volunteer. Potential participants were then provided with written information sheets to give them ample time to consider their participation. In the event that a person lacked or had begun to lose the capacity to give informed consent, the researcher was referred to a consultee and staff, drawing on their knowledge of that individual in making a decision on their participation. When obtaining initial consent, the participants were also asked for advance statements that set up their wishes and preferences with regards to continuing in the study and identifying a personal consultee should they reach a state in which they no longer had the capacity to decide for themselves. Obtaining consent took place as an ongoing process in ordinary interaction and communication (Dewing 2007). As Rachel’s dementia progressed, I reflected on her advance statement and her husband’s (the designated consultee) opinion.

The PhD project included residents with mild cognitive impairment (MCI) and early to moderate dementia who resided in two residential units in the Home (n = 17/87). However, residents without dementia who lived in the same residential units were also recruited if they wished to participate (n = 11/87). Other residents from two nursing and dementia units caring for residents with physical disabilities and advanced dementia were excluded (n = 84). Among those participants (n = 28), there were two couples in which one of the spouses had been diagnosed with a type of dementia. In this article I focus only on Rachel and Jacob so as to pay more attention to their affective creativity, rather than the other couple’s joint religious practice as orthodox Jews.

It is worth mentioning that this study is not representative, nor does it offer a theoretical alternative or specific clinical benefits; instead it calls for us to advance our understandings and discussions in relation to affective creativity in dementia beyond the currently predominant individual cognitive- and outcome- centered approaches. This ethnography of affective creativity thus focuses less on the quality of creativity, but more on the process, the situation and the ways in which the couple engages with the artworks.

It should also be mentioned that due to the conditions of residing in the same place at the same time, participants’ everyday practices are always and already intertwined and enmeshed with non-participants. In this sense, my fieldwork was collaborative in that participants as well as non-participants not only contributed to the research by way of co-presence and dwelling, but also performed their ordinary practices on their own terms and according to their own interests, and at their own speed (Fabian 2014). This ethnography is thus performative, reflexive and ethical, appreciating all subjects involved in the study including non-human beings, not only as participants but also as co-producers (Zeilig, West, and van der Byl Williams 2018; Swaffer 2016; Kontos et al. 2015). Consequently, although non-participants remain anonymous throughout the study, without addressing their co-presence and dynamic “transmission of affect” (Brennan 2004:3) I cannot demonstrate the whole lives of the participants. Such a situation calls for anthropologists to pay more attention to the affective dimension of ordinary ethics as a way of dwelling in care home settings.

The situations and ways in which co-producers make collaborative work vary, for example, depending on where the activity is taking place and what kinds of things are involved. The fieldwork may be either sedentary or mobile, either inside or outside, or both. Therefore, I used multiple methods. These included in situ and in-depth interviews with significant others during and after the activity. Collaborative life history was carried out not only to collect the biographical social life of the couple but also to explore how they create a joint story, alongside photo elicitation (e.g. Hydén 2017; Hydén and Forsblad 2017). All the collected data, including transcribed interviews and daily fieldnotes, then became ethnographic evidence used for analysis, emphasizing not only a joint account of the couple’s affective practices, but also their generative, situational, and relational meanings in the making of artworks.

## Contextualizing the Home and Arts Activities

Beginning life as three small Jewish poorhouses founded in the East End of London about 175 years ago, which were amalgamated at the end of nineteenth century and relocated to South London in 1904, the Home has undergone dramatic transformations in response to ever-changing social, cultural, economic, and political crises and demands.

Person-centered care (Kitwood 1997), implemented in partnership with Bradford University Dementia Group in 2011, was a tectonic shift from how the Home was operated in the 20th century with regard to the ways in which it organized medical, therapeutic, and psychosocial care. In consideration of the purposes of in-house clinics, it predominantly focused on ageing and functional (physical) health, in particular, mobility and body movement, in the first half of 20the century. By 1924, eye, ear and dental care facilities were made available in-house and by 1952, a chiropody department was established. Throughout the 1950s, physiotherapists practised some experimental treatments in the Home—for instance, faradism (electroshock for stimulating damaged or paralyzed muscles and nerves); infrared neural stimulation for boosting neural activity; and wax-baths for improving blood circulation and muscle relaxation and movement. However, electro-convulsive therapy used for anaesthetized residents had been highly controversial in terms of its benefits and side effects such as permanent memory loss, and thus was gradually no longer practiced by the 1960s. An occupational therapy department was first founded to provide advanced therapeutic practice (at the time) in 1949, such as making rugs, lampshades, handbags, wallets, stools, scarves, soft toys, lace dinner sets, bathmats, tray cloths, and baskets. Like other therapeutic treatments such practices were mainly used for maintaining, strengthening and rehabilitating body skills and movement. It was not until 1958 that the ‘weekly group musical circle’ became a regular programme whose psychosocial benefits was widely recognized in the Home. It is also worth mentioning that observing gender separation in the Orthodox Jewish tradition meant that man and women residents were not allowed to sit and do things together including wife and husband couples, and partners by early 1980s, though while today this tradition is still practiced in the on-site Orthodox synagogue, it is no longer practised in the Home.

The Home built the medical and therapeutic foundation of today’s care practice by the 1960s in terms of the numbers of qualified staff and on-site clinics. However, the ways in which staff members perceive, approach, and understand ageing and dementia did not changed much until the introduction of person-centered care. Indeed, the term “inmates” was widely used among staff members until a new four-storey building with 160 en-suite single rooms was built in 1976. Before the enactment of the new National Health Service and Community Care Act in 1990, the number of residents reached its peak of 430 with 350 care staff. The Act encouraged the elderly to stay in their own homes or communities as long as possible, so admission to newcomers has been even further delayed. Accordingly, there have been dramatic changes in the number and characteristics of residents in the Home. A decade ago, a dementia unit managed to handle all residents living with dementia (n = 40/200). As of 2015, over 80% of the residents had been diagnosed with a form of dementia. The average age of residents had already reached 90 by 2015, in comparison to 83 in 2000. These demographic changes have driven a fundamental transformation in care practices. Today, the Home provides individually tailored service with a full range of residential, nursing, dementia, respite and end-of-life care. The service includes not only medical and therapeutic care but also sociocultural, religious and arts activities, as seen below (Table 1). Due to limited space and the considerable body of data available, in this article I focus mainly on visual arts.

**Table 1:** Weekly Programmes (as of May 2015)

	Mon	Tue	Wed	Thu	Fri	Shabbat	Sun
AM	Arts Cooking Discussion Reading	Cooking Crafting Body exercise Arts	Cooking Knitting Arts Discussion	Crafting Arts Body exercise Reminiscence	Crafting Arts Flower arranging Discussion (Men)	Religious service	Family visiting
PM	French-group Discussion Music (Shopping)	Crafting Drama Discussion	Crafting Pet walks Film (Choir)	Crafting Tea party Bridge game Poetry	Crafting Bingo game Religious service	Tea & chat with a Religious leader	(Film)

## Affective Entanglement of the Couple

As Rachel’s dementia progressed, their couplehood and friendships have changed dramatically (e.g. Hellström, Nolan, and Lundh 2007; Hellström and Torres 2012; Eriksson, Sandberg, and Hellstrom 2013, Hellström, Eriksson, and Sandberg 2015). Rachel was barely able to do her daily routines, and Jacob thought seriously about finding a residential home where they could live and deal with her crisis together, near their house in Norfolk in the UK. However it was his son-in-law who recommended the Home, where his mother used to live. They moved into the Home in late 2013 at the cost not only of selling their house, in which their whole married life and memories are embedded and where their three children grew up, but also parting from their life-long friends, neighbors, and familiar landscape. It was a joint decision by the couple, even though “it wasn’t quite right” and “it wasn’t the best option,” as Jacob says. The Home is not just for the couple’s quality of care and life but, more importantly, they needed to consider that the move necessitates a long trip for their adult children and grandchildren to see them. Of course, it was not an easy decision to move into the Home for both Jacob and Rachel as Christians, because it is an Orthodox Jewish care home. Although Jacob was one of the children brought on the Kindertransport^1^ at the age of ten, he was raised in what he called a “totally professional” Quaker family, meaning that he needed to adjust to a strict Christian home discipline. Since then, he had completely lost his mother tongue and Jewish way of life, and had grown up as a “pure” Christian. Therefore living in the Home can feel “awkward” and “challenging” for the couple. Needless to say, the unexpected transition makes Rachel uncomfortable, and she often gets confused. “Are you reasonably happy?” their three grown children asked them one day. Rachel mumbled but Jacob clearly replied, “There won’t be happiness, but our lives go on.” Here the idea of “doing things together” becomes a central point around which to maintain couplehood by searching for things such as the “little pleasures, the positives, and living for today” (Hellström, Nolan, and Lundh 2007:394).

Jacob always thought he would leave this world before Rachel: after three heart operations, Jacob believed his heart could stop again any day, even any minute. Despite his fame as a local table tennis champion and the Chairman for the Table Tennis Society for a decade in Norfolk, he had to stop playing table tennis about two decades ago. Once he made the decision, he swiftly put it into action.

The couple therefore needs to find new ways of living in the unfamiliar place and environment. In particular, Jacob has realized that as Rachel’s dementia evolves, he needs to learn how to dwell with his wife’s conditions. These days, Jacob often witnesses Rachel struggling to converse with their grown-up children over the phone. There was a time when he tried hard to ‘fill in the gaps’ (Fontana and Smith 1989:39), in the sense that he normalized his wife by correcting her words, helping finish her sentences, and suggesting some words for her that she could use. This was his way of practising his care, love, and responsibility, which was often described as patronizing by the staff. This has changed over the past year. The more he tried to give (correct) her something, the more he lost. He has learned through trial and error and consultations with staff and specialists to stay connected while letting her go (Braff and Olenik 2005). Rather than correcting, rejecting, or normalizing Rachel’s utterances and behaviors (loss), he accepts and lives with them, just as they have lived through the past 60 years together (c.f. van Wijngaarden et al. 2018). Retaining hope that she might have a better day today, he suggests participating in artmaking which is one of the remaining activities they can do together. This suggests that the partners are regarded not just as a spouse, but as “my spouse” by “maintaining continuity; sustaining existing competencies; protecting the [spouse] from incompetence; strategizing public encounters; and ensuring that public appearances would be as comfortable” (Perry and O’Connor 2002:56–60). Interestingly, although what Jacob tries to do is for Rachel often described as informal or “technically a carer” by staff members, he hardly ever defines himself as a carer; rather he insists that it is a part of their relationship, his way of dwelling with his wife (Molyneaux et al. 2011:493).

In the art session, Jacob keeps his eyes on her at a distance, while making his own an artwork. This is Jacob’s way of going along with the here-and-now situation, supporting Rachel, and providing her with the necessary attention. He keeps cool, like he gets on with it while watching Rachel. He smiles. In Rachel’s room he continues to tell me:

Someone has had to cope with their life against an unusual and difficult start at the age of ten, and they are still fighting… But being with Rachel for more than sixty years, I think we cope very well and we will…

He then asks Rachel “Is that right? Say yes or no?” “Absolutely yes!” Rachel answers quickly. The conversation shows how the couple defines their relationship, more specifically how Jacob jointly perceives himself as a couple not only by referring to himself as a “we” (couple) rather than an individual, but also by positioning himself individually as “part of the we” (Hydén and Nilsson 2013:725). In addition, their more than 60 years of joint life enables Rachel to participate in the conversation. In other words, a such long-term relationship is not just a social unit, but works as a “scaffolding” which has been formed of mutually trust, endured life-long commitments, reciprocity and loyalty, and which is charged with a range of emotions (Hydén 2011:339; Merrick, Camic, and O’Shaughnessy 2013). Of course, for them, life has not always unfolded as they expected; rather it is contingent and full of ups and downs.

For Jacob, nothing could be worse than during the War when his entire family ended up at Auschwitz without any chance to say farewell. Long after the War, he visited his home town in Austria a few times and met some surviving relatives, but these relations did not develop further. As a 10-year-old boy, there was nothing he could do at the time except calmly and steadily adjust to the unforeseen world and strengthen his internal and physical stability for the future. As soon as he could start to work, he tried to stand on his own two feet. He began to work in a local shop when he was about 15 years old, and he continued to study at an art school where he met Rachel, and they both became architects. However, their married life has not always gone smoothly. They had to wait almost 5 years for their first child, trying everything they could, including travelling from Norfolk to London to visit a specialist. They did not have enough money to buy a house, so they bought land on which to build their house, a project on which they worked every weekend, and which took them almost 10 years to complete. Retrospectively, Jacob emphasizes that in time, there would be a time for everything but, until then, they needed to try their best not to become frustrated or give up.

Rachel gave up drawing and painting before entering the Home. Since her late fifties, she has been living with depression. As their children married and formed new families, she thought she could enjoy the rest of life with Jacob. She began to draw and paint in earnest. In her seventies, however, her depression became severe and her memory was often unclear. This was the one of reasons that Rachel had stopped doing art a long time ago, when she first realized that she was losing her artistic techniques and skills, and before she was formally diagnosed with dementia. Jacob no longer asked or recommended that she did art either.

A new life brings new friends. They were invited to the arts session by activity leaders and other residents. In particular, Yiskah (87) (without dementia), who is also one of the Kindertransported children, is one of those who encourage the couple to join diverse activities. Yiskah, who is culturally Jewish, often invites Rachel to her art world as a portrait model, while Rachel engages in her work. The couple becomes regular artists in the Home, making new friends. Furthermore, Yiskah’s curiosity about Jacob’s total loss of youth memories to some degree stimulates Jacob to explore his forgotten Jewishness. These days they continue to muddle through together, wrestling against adversity. They know it will not be the last. I asked what changed for them that they both felt ‘comfortable’ or restarted doing art again and what would the couple do next then? He says:

The future… it is what it is now. If we like it, we will stay longer, for one year or maybe five… But I do not have a future. Maybe the children, but not us! We are just facing forwards… Look at the pictures on the wall. She did it all but now she does not have it anymore…

For the couple, it is never too late to start to learn new things, opening a new chapter in their joint life.

As a couple, they tried to be perfect in terms of the way they distributed and shared their family histories and memories. Rachel played a significant role in memorizing family anniversaries, such as birthdays and celebratory events, keeping all the information in her memory; while Jacob did practical things, such as organizing events and collecting material evidence, such as photographs. Now, things have completely changed. It is “a miracle” that he still has a scrapbook made of photographs and cuttings published in local newspapers and magazines about his family life, including pictures of him with table tennis trophies. When he started making the scrapbook, it was just for fun and to support his memories, but it has become essential both to his own memory and to stimulating his wife’s memory.

In brief, such transformative couplehood indicates “the dynamics of dementia” in ways of “working together, working separately, working alone or working apart” (Keady and Nolan 2003). But their relationships should not be perceived from a caregiver’s perspective alone; rather it calls for a relational view, emphasizing the ways in wich the couple co-construct couplehood (Merrick, Camic, and O’Shaughnessy 2013; Hellström, Nolan, and Lundh 2005, 2007; Graham and Bassett 2006). In other words, their couplehood and their “us identity” (Davies 2011:128) are not based on a one-way dependent or caring relationship; rather they continuously emerge and are shaped by (and shape) social processes (Perry and O’Connor 2002). As Jacob’s failure to fill her gaps demonstrates, Rachel is not only “socially responsive but actually sometimes actively structure[s] [her] relationships with [her] spouse in quite complex ways” while at the same time, for Jacob, it is his “challenge to create a distance to strong We[ness] with [Rachel] if there is a need for that” (Hydén and Nilsson 2013:730) for instance, he had to wait a year to get a separate room from Rachel. Likewise, participating in arts activities should be read as a co-construction that the couple not only seek for maintaining their couplehood, but also for learning and creating the possibility of becoming otherwise, while dwelling with and through dementia.

## Distributed Collaborative Creativity

As Rebecca (an activity leader) mentions, the ways in which staff members organize and facilitate activities vary depending on the different needs, medical conditions and preferences of individual residents, and on the available resources at any particular time and place. In this sense, arts activity is ambiguous in that individual participants have different interests and tastes, and therefore tailored initiatives are preferable when it comes to facilitating these activities. More importantly, due to the fluctuations and different forms of dementia, potential participants are always indeterminate not only in terms of the number of participants, but also in terms of their particular characteristics, as they all have different requirements and react differently toward here-and-now social and material circumstances. Indeed, activity leaders cannot control the criteria for participants, nor can they anticipate exactly who would join the activity, or predetermine which materials they would be able to use.

Arts activity is improvisational not because residents with dementia are living in the past, but because there are no predetermined forms, destinations or directions they should follow. For example, as Elena, an activity leader, discusses in her farewell letter:

My plan had originally been to paint several watercolors of singular objects belonging to the residents, items that reflected their personality in some way and could pose as a form of portrait. After my first meeting with Elaine I realized that the residents not only did not have many belongings but did not need or react to objects in the same way that they used to, other than the odd teddy and bundle of knitting. My plan evolved to more of an imaginative realm whereby the residents considered images, places, colors, animals and objects that they wanted to see in the painting.

In addition, activity leaders are not only contributing their skills and ideas to arts activities: they also support, stimulate and motivate residents’ creative powers by acting as facilitators and building a stimulating atmosphere. Knowing the background of individual residents, such as their life history, career, personality, preferences and medical history, is extremely helpful for improving and strengthening friendly interaction and communication. For example, Rachel needs ample room to adjust to her illness on her own terms and at her own speed. Rachel may not respond to the drawing at first without Jacob’s initial encouragement. As Claudia (an activity leader) says, Rachel is one of the most difficult residents to explain something to in a way she can understand due to her loss of cognition and short-term memory. Rather than explaining how to do something once, Claudia instead shows the processes of artmaking step by step, and helps Rachel to work in her own way.

In this sense, as Fiona (an activity leader) insists, artmaking is collaborative and improvisational in that the ways in which activities are organized and facilitated for both staff and residents is based on here-and-now situations, interactions, and communications. Claudia adds, “For Rachel, it was not about the drawing but about the color. That gave her something, although not confidence. But at least she could do something which didn’t have to be exact, but she could focus on it.” This is in contrast to her husband’s early interventions to assist and help her by explaining, showing and demonstrating what she wants to do and say, as this gave her no room to adjust to her world of dementia. Clearly, these requirements do not have universal and one-off unique solutions. Solutions need to be responsive and attuned to specific situations. In particular, the provision of a wide range of activities requires not only the “emotional labour” of the staff (Adams 2007:191), but has the effect of diversifying their skills, experiences and human relations in a collaborative way. For example, over the past decade Fiona’s career as an activity leader has extended into knitting, storytelling, singing, befriending and almost everything and everywhere that people with dementia reside. She does not need special tools or particular media in order to facilitate an activity. All ordinary things, stories, encounters and lives are indefinite resources for her, a reservoir of improvised creativity. Fiona told me:

My husband had dementia for 7 to 8 years. I always engaged with him in terms of his capacity and preferences. We peeled carrots endlessly because he wanted to do something. What he used to like to do was mending things, but these skills were gone. But he wanted to be with me and cooking with him was quite good, although I had to eat carrots for a while. Similarly, you have to know a little bit of biography and life history. Someone likes doing this, others don’t… interacting and finding out…

From her point of view, to provide quality of care and life is not an unreachable goal, but is already and always here around us, we just need to realize it. Interestingly, the way she works with people with dementia relies very much on her personality and previous experiences with her husband, who went through dementia. Recently, she has reduced her working hours and increased her voluntary work in the Home and with the Alzheimer’s Society in London. As she said, it was the experience, know-how and skills gained during these difficult times that meant she was able to give back to society. The basic concept of organizing such an activity is “person first” in her words, taking into account “what they can do” and “what they want to do” first. In this way, even vulnerable people who have lost their abilities can participate in artmaking in a collaborative way. As Claudia says, it is a “communal effort” in that the staff do not see individual limits, instead seeing the immanent potential of becoming otherwise that emerges through interaction and encounter.

Arts activity is relational, and taking into account how every resident is already situated in their immediate biosocial surroundings requires us to acknowledge the enmeshed relations and interactions between people, things and environments. For example, at the beginning of a crafting session, calm, classical music might be playing, although pop or jazz music from the 1930s and 40 s may also be used. As time goes on, increasingly rhythmic and livelier music is played, while the smells from the kitchen and the flowers, the circulation of fresh air, the adjustment of sunlight and room temperature are also attuned to contribute towards a dementia-friendly atmosphere. At the same time, it is essential for activity leaders to arrange the seating and work-partners of those affected as, depending on whom they work with, they will respond and act in significantly different ways to the session. Rachel does not much care whom she sits by so long as it is a relatively calm and kind person. Indeed, residents do not only practice their artistic skills in the center, they also navigate, constitute and experience these kinds of affective and sensory “atmospheres” (Ingold 2012a, b).

In brief, arts activity is experienced and practised in the process of collaborative and participatory engagement with staff, residents, materials and physical environments. From a staff point of view, organizing and facilitating such activities requires them to “collaborate more productively” not only with the material, but also with their own and residents’ cultural, historical and medical legacies, and the atmospheric environment (Ingold 2013:31). Although they work individually by distributed assignment, their participation in arts activities always make them collaboratively interwoven through interacting with one another beyond human and nonhuman boundaries. These open-ended and enmeshed relations are embedded in a particular socio-cultural, historical, and environmental context which opens up the potential possibilities of dementia-becoming in the making of artworks.

## The Affective Social Life of Rachel’s Artworks

Rachel’s loss in language, body functions and memory generates and not only stimulates her idiosyncratic ways of expressing and representing her world of dementia through diverse colors and repetitive patterns. It also affects the subsequent transformation of the couple’s relationship and the ways they live out difficult moments. At first, many people in the Home said that Rachel’s skills and dexterity have gradually deteriorated in one way or another, but her newly emergent modes of expressive performativity lead to different aesthetic interpretations amongst the people around her. As activity leaders claim, her passion for engaging in artmaking is deeply embedded not only in her aesthetic sense but also in the affective, sensory, and expressive dimensions of the couple’s life. It is a corollary here that the question I attempt to explore is less about discovering how far her participation in artmaking can improve her health and well-being, but more about exploring the ways in which couplehood affect and is affected in the making of artworks, and how she expresses her world of dementia within the affordance of her illness, as an affective and creative self.

**Fig. 2:**
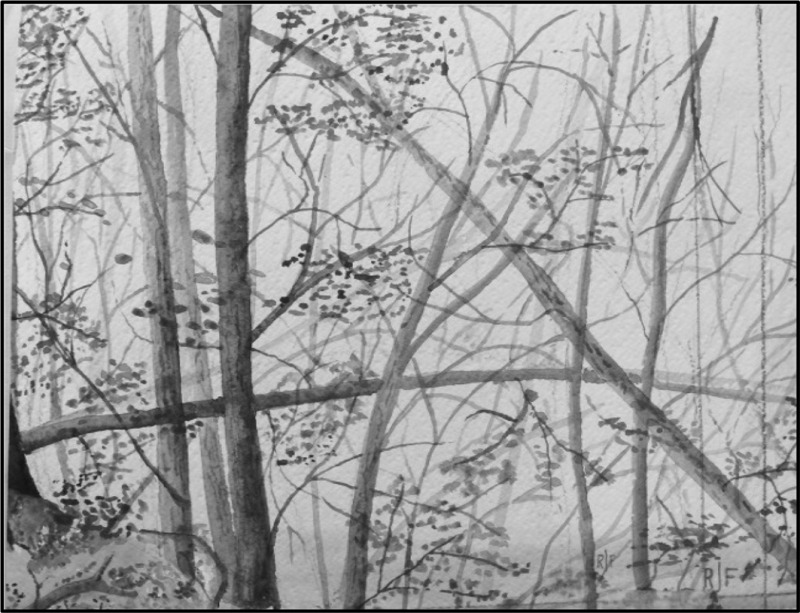
Rachel’s Painting before Dementia

Jacob always admired Rachel’s delicate, detailed paintings and drawings before the onset of her dementia (Fig. 2). Like his scrapbook, he has proudly kept a portfolio of her artworks through her life and some pieces hang on the wall in her room in the Home. Meanwhile, the recent dramatic changes in her artworks mean that sooner or later, she will no longer have a “clear mind.” For Jacob, the artworks he looks at now are thus not mere “art objects” that represent Rachel’s “aesthetic response” or symbolic “meaning” (Gell 1998:5), nor are they just the “neurology of art” (Mendez 2004) that enable him to identify the neurological deterioration of her brain. Instead, these artworks offer one of the few means left for the couple to communicate with each other. In addition, because of their dramatic joint life stories, her artworks are invaluable beyond her illness and her aesthetic performativity. Here, time has multiple dimensions in social life. It is not like our usual means for counting, calculating and measuring, but embraces the multiple qualities and implications of the couple’s emotional, moral and biographical life. This draws my attention to the work of Binaca Brijnath. In her book *Unforgotten* (2014) she demonstrates that although there is socially and culturally embedded prejudice and stigma around dementia and caring for those affected, concomitantly we always have possible ways of showing, expressing and performing our love. Brijnath (2011) shows how Tandon, a husband, cares for Sheila, his wife, living with advanced dementia in India. Although Sheila hardly responds to any stimulation during the daytime, she shows her emotional expressions when she is offered ice cream early in the morning. Feeding ice cream to his wife at 3am becomes not only a daily routine, but also his own way of demonstrating care and love. Likewise, from Jacob’s point of view, participating in artmaking is perhaps one of the remaining ways he is able to not only show his feelings of love and attention, but also to do things together, although it also demands a sense of loss and frustration in the recognition that nothing can be done for his wife, he can only stand by her (e.g. Gillies 2012).

Of course, the couple’s journey has not stopped here. Jacob, as well as other activity leaders have tried to offer Rachel diverse arts activities (e.g. drawing, crafting, cutting, painting, walking, cooking, arranging flowers, appreciating artworks, and befriending pets such as cats, dogs, rabbits and guinea pigs, o name a few) with different tools (e.g. clay, color paint, fresh leaves and flowers and so on). These have become what Janelle Taylor (2017:286) calls “moral experiments,” adopted from Cheryl Mattingly’s (2014) “moral laboratories.” Rachel is continuously encouraged to engage in activities so as to keep her relationships and interactions flowing in the adversity of her dementia, recognizing and enhancing her “habituated, embodied or emotional” capacity (Boyle 2014:1132).

## Conclusion and Implications

Focusing primarily on feeling bodies and the affective dimensions of artworks in the Home, I have reconceptualized this bodily affective performativity not as a mere pathological biomarker but as a differentiating force that continually brings something into being through the process of everyday practice. Unlike previous methods which are mainly researcher-led and pre-designed, this project focuses on day-to-day experience as a research method, conducting a collaborative work with residents and their significant others. This research thus contributes not only to critically developing perceptions and understandings of the affective creativity of people living with dementia—in particular, for couples—but also to offering new ethical, theoretical, and methodological frameworks in the study of dementia from an anthropological perspective.

The couple experiences artmaking in significantly different ways, not only because of their idiosyncratic biographies and personalities, and Rachel’s dementia, but also because of the ways the Home organizes and facilitates activities in response to ever-changing individual situations and available social and material resources. More importantly, activities are experienced and facilitated not as mere individual aesthetic activities, but rather as “creative entanglements” among people, things, and environments (Ingold 2010). These activities thus are characterized as improvisational, relational, and collaborative. They are improvisational in that no original plan works as expected. Continuous improvising is needed in response to ever-changing immediate situations. There is no predesigned script to follow (Hallam and Ingold 2007). The activities are relational, referring to the way staff work—particularly when arranging seats and talking to residents—to residents’ relations with working partners as well as their biographies, personal preferences, personalities, and the nature of their illness. They are collaborative in the sense that staff members, as well as the couple, do not see an artwork as an isolated single activity or an individual task, but rather as something which is made up of the entangled interactions and encounters with the immediate surroundings.

Consequently, this ethnography reveals that the foundation of the couple’s affective creative force is different from other artworks across illness contexts in terms of their interdependency and vulnerability as a result of the illness. As Rachel undergoes different levels of dementia, her works are continuously transformed. It is fair to say that approaching her works requires a fundamentally different approach and understanding in terms of not only aesthetics, but also of the ways in which the couple engages with artworks. It is true that Rachel to some extent lacks self-sufficiency due to her vulnerability and dependency. However, through her artworks, she participates in collaborative creativity, not only by using her embodied techniques and skills, but also by sharing and distributing her affective practice, which is both bodily and socio-culturally embedded in her body. For Rachel art is the means by which she can now communicate with her surroundings, and more importantly with her family. At the same time, the process of her engagement with her artworks is a co-affective journey with her husband. However, because of her illness and Jacob’s own medical conditions, their journey is uncertain and unsteady. This day could be their last but, hopefully, they will have many more, and so artworks for them are not just about aesthetics, but are also a medium through which to communicate, and an affective testimony by which they pass through the here-and-now.

Concomitantly, a new form of affective creativity emerges through continuous interaction and encounters responding to the here-and-now personal, social and material situations. In this regard, I share Ingold’s (2010) critique of the recent disproportionate concern over completed products, their usages and circulations, which result in neglecting the actual process of making. In *Making and Growing* (2014:xiii), Hallam and Ingold insist that our understanding of materials, design, and creativity should focus not so much on the relations between makers and the finished objects, but more on the complex and entangled process of the “becoming of persons” and “becoming of things.” The trajectory of artworks is open and involves more rhizomatic becoming, in that they are enmeshed in entangled relations where there is neither a start nor an end, but in Deleuze and Guattari’s sense (2004) they keep generating something in unpredictable ways. Here, participating in arts activities reveals a potential site for those “to affect and be affected” in the way that “one always affects and is affected in encounters… through events” (Massumi 2015:ix), producing “co-creativity” (Zeilig, West, and van der Byl Williams 2018:138).

Finally, this study suggests affect as a distributed creative force and a new modality of dementia-becoming by revealing a continuous process of individual affective differentiation and transformation over the course of the dementia trajectory. It allows us to explore the affective dimension of the everyday creativity of couples whilst people living with dementia continue with their mundane lives, minimizing unnecessary intervention and maximizing social inclusion and engagement regardless of mental and physical capacity. Detailed ethnographic findings will offer a better care practice which considers how dynamic and complex affective performativity can improve creativity, and be used in dealing with personal crises in everyday life. Finally, in order to fully understand the complexities and potential possibilities of affective creativity, the study needs to be extended towards different types of activities, dementia, people, and contexts.

## Footnotes

1 The *Kindertransport* was a unique British humanitarian rescue mission that evacuated approximately 10,000 children from Nazi-occupied territory in Europe just before the outbreak of the Second World War.
